# Synthesis and Antitumor Evaluation of 6-Aryl-substituted benzo[*j*]phenanthridine- and Benzo[*g*]pyrimido[4,5-*c*]isoquinolinequinones

**DOI:** 10.3390/molecules171011616

**Published:** 2012-09-28

**Authors:** Jennyfer Iribarra, David Vásquez, Cristina Theoduloz, Julio Benites, David Ríos, Jaime A. Valderrama

**Affiliations:** 1 Facultad de Ciencias de la Salud, Universidad Arturo Prat, Casilla 121, Iquique 1100000, Chile; 2 Facultad de Ciencias Químicas y Farmacéuticas, Universidad de Chile, Santiago 8380492, Chile; 3 Instituto de EtnoFarmacología (IDE), Universidad Arturo Prat, Casilla 121, Iquique 1100000, Chile; 4 Facultad de Ciencias de la Salud, Universidad de Talca, Talca 3460000, Chile; 5 Facultad de Química, Pontificia Universidad Católica de Chile, Casilla 306, Santiago 6094411, Chile

**Keywords:** *N*-heterocyclic quinones, enaminones, cytotoxicity, SAR analysis

## Abstract

A variety of novel 6-arylsubstituted benzo[*j*]phenanthridine- and benzo[*g*]-pyrimido[4,5-*c*]isoquinolinequinones were synthesized from 1,4-naphthoquinone, aryl-aldehydes and enaminones via a two-step synthetic approach. The cytotoxic activity of the aminoquinone derivatives was evaluated *in vitro* against one normal cell line (MRC-5 lung fibroblasts) and three human cancer cell lines (AGS human gastric adenocarcinoma; SK-MES-1 human lung cancer cells, and J82 human bladder carcinoma) in 72-h drug exposure assays using the MTT colorimetric method. Structure–activity relationships within the series of angular quinones reveal that the insertion of pyrrol-2-yl and furan-2-yl groups at the 6-position is more significant for the increase of the potency and selectivity index of the pharmacophores.

## 1. Introduction

The quinone nucleus is common to many natural and synthetic products associated with anticancer and antibacterial activities [1]. Among these compounds the polycyclic members are typically DNA-intercalating agents due to the ability of their large and planar structure to bind strongly between the base pairs through hydrogen bonds and *π*-stacking interactions [2,3]. They usually have side chains or sugar substituents and basic nitrogens, which upon protonation further strengthen the DNA binding. Examples of quinone derivatives with antitumor activity include mitoxantrone [4], doxorubicin [4], mitomycin [5], streptonigrin [6] and actinomycin D (AMD) [2]. All known quinonic DNA intercalators have the potential to disrupt the normal function of DNA, leading to cell death [7]. This DNA damage can be caused either by the parent form or by its metabolic conversion to electrophilic or radical species [8].

**Figure 1:**
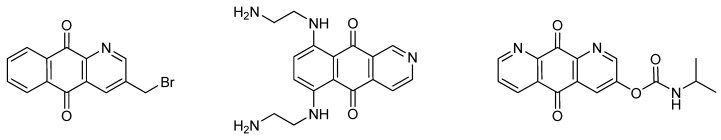
Examples of aza-anthraquinones with potent antitumor activity.

Aza- and diaza-anthraquinones such as those depicted in Figure 1, represent an important class of antitumor agents that exhibit promising *in vitro* and *in vivo* activity on tumor cell lines [9,10,11,12,13]. The antitumor activity of these agents seem to be mediated by DNA intercalation and redox cycling processes that are improved by the basic and electron-withdrawing properties of the *N*-heterocyclic ring [12].

**Figure 2:**
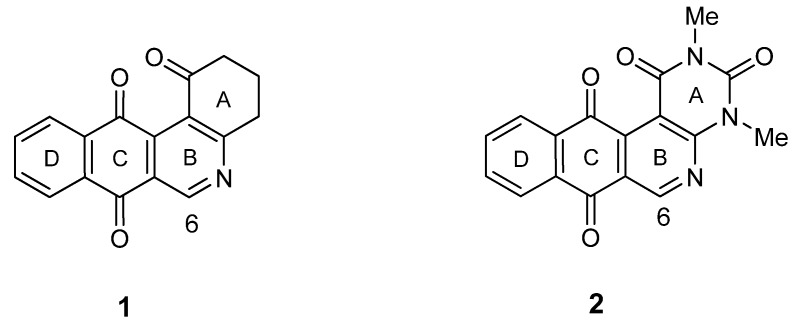
Structure of angular *N*-heterocyclic quinones with antitumor activity.

As part of a research program on the synthesis and antitumor evaluation of *N*-heterocyclic quinones we have described the synthesis and antitumor properties of benzo[*j*]phenanthridine- and benzo[*g*]-pyrimido[4,5-*c*]isoquinolinequinone derivatives [14]. Recent evidences demonstrate that some members of these series have potential anti-cancer activity through inhibition of TOP1 catalytic activity and induction of apoptosis [15]. The synthesis of these tetracyclic quinones, such as **1** and **2** (Figure 2), was accomplished by means a strategy involving the heterocyclization of acyl-1,4-benzoquinones with enaminones to construct the ABC-ring systems followed by a Diels-Alder reaction to assemble the D-benzene nucleus. 

In this context, we envisaged an alternative synthetic approach to the tetracyclic frameworks, in which the assembly of the ABCD-ring system would be accomplished through a one-step process by heterocyclization of 2-acyl-1,4-naphthoquinones with enaminones. Taking advantage of recently published preliminary results on the synthesis of heteroaroylnaphthohydroquinones by solar-chemical photo-Friedel–Crafts heteroacylation of 1,4-quinones [16], we decided to explore the mentioned strategy. Herein, we wish to report the synthesis and the *in vitro* antitumor evaluation of a variety of 6-aryl-substituted benzo[*j*]phenanthridine- and benzo[*g*]pyrimido[4,5-*c*]isoquinolinequinones. In the light of the results, structure-activity relationships (SARs) were examined, using **1** and **2** as lead compounds, assessing the effects on antitumor activity of structural changes made to the pyridine fused ring B by insertion of aryl- and heteroaryl groups.

## 2. Results and Discussion

### 2.1. Chemistry

**Scheme 1:**
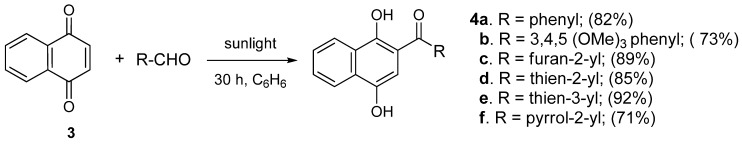
Preparation of acylnaphthohydroquinones **4a**–**f**.

Acylnaphthohydroquinones **4a**–**f** together the enaminones 3-aminocyclohex-2-en-1-one (**5**) and 5-amino-1,3-dimethyluracil (**6**) were selected as precursors to explore the synthesis of the designed *N*-heterocyclic quinones. The synthesis of the required hydroquinones **4a**–**f** was performed by solar photo-Friedel-Crafts reaction of 1,4-naphthoquinone (**3**) with the corresponding aryl- and heteroarylcarbaldehydes, according to our recently reported procedure [16]. In all the examined cases acylhydroquinones **4** were obtained in good to excellent yields (Scheme 1). The structure of compounds **4a** and **4c**–**f** were confirmed by comparison of their spectral data to those of authentic samples [16]. In the case of the previously unreported compound **4b**, its structure was established by ^1^H-, ^13^C-NMR, and HRMS data.

**Scheme 2:**
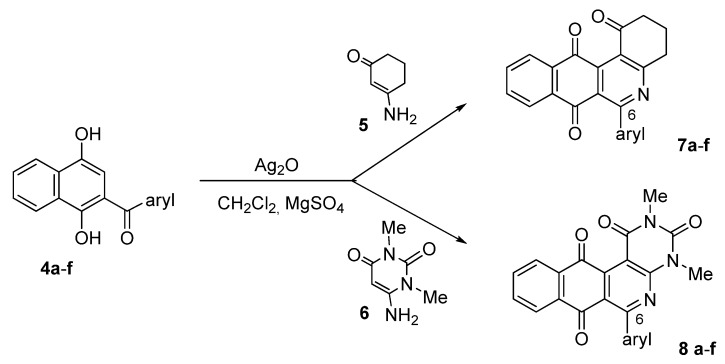
Synthesis of 6-substituted angular quinones **7** and **8**.

The synthesis of the target members of the series of benzo[*j*]phenanthridine- and benzo[*g*]pyrimido[4,5-*c*]isoquinolinequinones **7** and **8**, containing phenyl and heteroaryl substituents at 6-position, was accomplished by reaction of acylnaphthohydroquinones **4**, enaminones **5**/**6** and silver (I) oxide in dichloromethane (Scheme 2) using a previously reported one-pot procedure [14]. The broad application of this synthetic pathway allowed us to synthesize derivatives with different aryl-substituents in position 6 of the heterocyclic ring systems. The results are summarized in Table 1.

### 2.2. Cytotoxic Activities

Compounds **7a**, **7c**–**f**, **8a**–**f** were evaluated for *in vitro* anticancer activity against normal human lung fibroblasts MRC-5 and three human tumor cell lines: AGS gastric adenocarcinoma, SK-MES-1 lung, and J82 bladder carcinoma, in 72-h drug exposure assays. The cytotoxicity of the new compounds was measured using a conventional microculture tetrazolium reduction assay [17,18,19]. The broad variety of the synthesized compounds was designed in order to gain insight upon the influence on the biological activity of phenyl, 3,4,5-trimethoxyphenyl, furan-2-yl, thiophen-2-yl, thiophen-3-yl, and pyrrol-2-yl groups at the 6-position of the benzo[*j*]phenanthridine- and benzo[*g*]pyrimido[4,5-*c*]isoquinolinequinone pharmacophores. The cytotoxic activities of compounds **7** and **8** are collected in Table 2. The cytotoxic activity of analogues **1** and **2** [14] is also included in Table 2 in order to compare the effect of the insertion of aromatic groups at 6-position of the corresponding chromophores. As indicated in Table 2, the tested compounds showed moderate to good activity in the *in vitro* antitumor screening expressed by the IC_50_ values. The novel angular quinones are less cytotoxic than the anti-cancer agent etoposide used as reference.

Comparison of the cytotoxic potency of the previously reported benzo[*j*]phenanthridine- and benzo[*g*]pyrimido[4,5-*c*]isoquinolinequinones **1** and **2** with the 6-aryl-substituted analogs **7a**–**f**/**8a**–**f**, evaluated in this study, clearly indicates that the biological activity on normal and human cancer cells depends on the nature of the substituent located at the 6-position. 

The initial structure-activity relationship (SAR) was focused on the effects of insertion of aryl-substituents at the 6-position of the benzo[*j*]phenanthridinequinone chromophore (compound **1**). According to the IC_50_ values it can be deduced that the insertion of a phenyl group in the 6-position of **1**, as in quinone **7a**, does not induce a significant change on the cytotoxic activity and selectivity index (SI = IC_50_ fibroblasts/IC_50_ cancer cells) on human gastric adenocarcinoma cell line. However, the substitution strongly decreases the biological effect, respect to **1**, on human lung cancer and bladder carcinoma cell lines.

Comparison of the cytotoxic potency between the furan-2-yl-substituted quinone **7b** and **1**, reveals that insertion of the furyl group at the 6-position results in a decreasing effect on the potency on human gastric adenocarcinoma cells and the suppression of the cytotoxic activity on normal lung fibroblasts, lung cancer and bladder carcinoma cell lines. The insertion of a thiophen-2-yl or thiophen-3-yl substituent, bonded at the 6-position of the pharmacophore, as in compounds **7c** and **7d**, promotes an increasing effect on normal lung fibroblasts and no significant changes on the cytotoxicy on the tested cancer cell lines.

The data of Table 2 reveal that on AGS cells analogue **7e** shows higher antitumor potency but a similar selectivity index to compound **1**. Also observed is that the insertion of the pyrrol-2-yl substituent suppresses the cytotoxic activity on lung cancer and bladder carcinoma cell lines.

We can conclude that among the evaluated 6-arylbenzo[*j*]phenanthridinequinones, the pyrrolyl-derivative **7e** could be of interest for the design of new analogues with antitumor activity on human gastric adenocarcinoma cells.

Next, the SAR analysis was focused on the effects of the insertion of aryl groups at the 6-position of the benzo[*g*]pyrimido[4,5-*c*]isoquinolinequinone scaffold (compound **2**). The data in Table 2 reveal that the insertion of a phenyl group in the 6-position of **2**, as in quinone **8a**, has a remarkable effect on the antitumor potency and selectivity index of all the tested cancer cell lines. It can be seen that the effect of such insertion on the cytotoxic activity on gastric and bladder cancer cells, reaches values nearly 18- and 16-times higher than the reference compound **2**. Compound **8b** was included in the cytotoxic evaluation on the basis of the biological activity of **8a** and precedent structure-activity relationships of benzo[*c*]phenanthridines, which reveal that methoxyphenyl substituents lead to derivatives with enhanced antiproliferative activity on cancer cells [20] respect to the unsubstituted phenyl-derivative. According to the biological evaluation of **8b**, the expected enhanced effect respect to compound **8a** was not observed. On the contrary, compound **8b** displayed significant less cytotoxic activity on the tested cell lines, compared to **8a**.

Next we examined the influence on the insertion of heterocyclic groups in the 6-position of the benzo[*g*]pyrimido[4,5-*c*]isoquinolinequinone chromophore. The substitution of a furan-2-yl group, as in **8c**, resulted in the suppression of the antitumor activity on lung cancer and bladder carcinoma cell lines but in a 25-fold increase in potency on gastric cell line compare to compound **2**, with a high selectivity index (9.6). The insertion a thiophenyl substituent, bonded through the 2- or 3-position to the 6-position of the chromophore, as in compounds **8d** and **8e**, does not induce significant changes on the cytotoxicy of the tested cancer cell lines. Concerning the effect of the pyrrol-2-yl group, as in **8f**, a strong decreasing effect on the cytotoxic activity of all the tested cell lines was observed. 

## 3. Experimental 

### 3.1. General

All reagents were commercially available reagent grade and were used without further purification. Melting points were determined on a Stuart Scientific SMP3 apparatus and are uncorrected. The IR spectra were recorded on an FT Bruker spectrophotometer using KBr disks, and the wave numbers are given in cm^−1^. ^1^H-NMR spectra were run on Bruker AM-200 and AM-400 instruments in deuterochloroform (CDCl_3_). Chemical shifts are expressed in ppm downfield relative to tetramethylsilane (TMS, δ scale), and the coupling constants (*J*) are reported in Hertz. ^13^C-NMR spectra were obtained in CDCl_3_ at 50 and 100 MHz. Chemical shifts are reported in δ ppm downfield from TMS, and *J*-values are given in Hertz. HRMS were obtained on a Thermo Finnigan spectrometer, model MAT 95XP. Silica gel Merck 60 (70–230 mesh) was used for preparative column chromatography and TLC aluminum foil 60F_254_ for analytical TLC. Acylnaphthohydroquinones **4a**–**f** were prepared according to a recently reported solar photoacylation procedure [16]. The structure of **4a**, **4c**–**f** were confirmed by comparison of their spectral properties with those of authentic samples.

*(1,4-Dihydroxynaphthalen-2-yl)(3,4,5-trimethoxyphenyl)methanone* (**4b**). This compound was prepared in 60% yield from 1,4-naphthoquinone (**3**, 500 mg; 3.16 mmol) and 3,4,5-trimethoxy-benzaldehyde (620 mg, 3.16 mmol); orange solid, m.p.: 189–191 °C. IR (KBr): ν_max_ cm^–1^: 3463 (OH), 1579 (C=O). ^1^H-NMR (CDCl_3_ + DMSO-*d*_6_): δ 3.93 (s, 9H, 3 × OMe), 7.03 (s, 2H, 2′ - and 6′ -H), 7.12 (s, 1H, 3-H), 7.56 (t, 1H, *J* = 7.6 Hz, 6- or 7-H), 7.66 (t, 1H, *J* = 7.6 Hz, 7- or 6-H), 8.18 (d, 1H, *J* = 8.0 Hz, 5- or 8-H), 8.43 (d, 1H, *J* = 8.4 Hz, 8- or 5-H), 9.25 (s, 1H, OH), 13.38 (s, 1H, OH); ^13^C-NMR (CDCl_3_ + DMSO-*d*_6_): δ 55.8 (2 × C). 60.3, 106.4 (2 × C), 106.6, 111.3, 121.9, 123.7, 125.3, 125.7, 128.9, 129.5, 133.0, 140.4, 144.1, 152.3 (2 × C), 156.6, 199.2. HRMS (M^+^): *m/z* calcd for C_20_H_18_O_6_: 354.11034; found: 354.10955.

*General procedure for the synthesis of 6-aryl-substituted benzo[j]phenanthridine- and benzo[g]pyrimido[4,5-c]isoquinolinequinones*: A suspension of **4**, the respective enaminone **5** or **6**, silver (I) oxide, anhydrous MgSO_4_ (0.3 g) and dichloromethane (20 mL) was vigorously stirred at room temperature after completion of the reaction as indicated by thin layer chromatograpy (TLC). The mixture was filtered through celite, thoroughly washed with CH_2_Cl_2_ and the filtrate was evaporated under reduced pressure. Column chromatography of the residue (70:30 petroleum ether/ethyl acetate) yielded pure samples of the corresponding *N*-heterocyclic quinone.

*6-Phenyl-3,4-dihydrobenzo[j]phenanthridine-1,7,12(2H)-trione* (**7a**). Prepared from **4a** (50 mg; 0.19 mmol), enaminone **5** (42.1 mg; 0.38 mmol), Ag_2_O (396.9 mg; 1.71 mmol); (4 h, 30.1 mg, 45%), yellow solid m.p.: 250–252 °C. IR (KBr): ν_max_ cm^–1^: 1676 (C=O), 1665 (C=O). ^1^H-NMR (400 MHz, CDCl_3_): δ 2.31 (q, 2H, *J* = 6.4 Hz, 3-H), 2.97 (t, 2H, *J* = 6.4 Hz, 2-H), 3.21 (t, 2H, *J* = 6.4 Hz, 4-H), 7.49 (m, 5H, arom), 7.79 (m, 2H, 8- and 9-H), 8.08 (m, 1H, 7- or 10-H), 8.15 (m, 1H, 10- or 7-H). ^13^C-NMR (100 MHz, CDCl_3_): δ 21.59, 33.28, 39.19, 125.64, 126.87, 127.16, 127.96, 128.23 (2C), 128.59 (2C), 129.25, 133.57, 134.12, 134.31, 134.53, 140.26, 144.53, 163.32, 166.74, 182.05, 183.77, 197.95. HRMS (M^+^): *m/z* calcd for C_23_H_15_NO_3_: 353.10519; found 353.10471.

*6-(3,4,5-Trimethoxyphenyl)-3,4-dihydrobenzo[j]phenanthridine-1,7,12(2H)-trione* (**7b**). Prepared from **4b** (50 mg; 0.14 mmol), enaminone **5** (78.40 mg; 0.71 mmol), Ag_2_O (163.48 mg; 0.71 mmol); (1 h, 58.1 mg, 93%), yellow solid m.p.: 243–244 °C. IR (KBr): ν_max_ cm^–1^: 1708 (C=O), 1675 (C=O). ^1^H-NMR (400 MHz, CDCl_3_): δ 2.31 (q, 2H, *J* = 6.4 Hz, 3-H), 2.96 (t, 2H,, *J* = 6.4 Hz, 2-H), 3.22 (t, 2H, *J* = 6.4 Hz, 4-H), 3.88 (bs, 6H, 3′ - and 5′ -OMe), 3.94 (bs, 3H, 4′ -OMe), 6.72 (bs, 2H, 2′ - and 6′ -H), 7.81 (m, 2H, 8- and 9-H), 8.09 (m, 1H, 7- or 10-H), 8.16 (m, 1H, 10- or 7-H); ^13^C-NMR (100 MHz, CDCl_3_): δ 21.58, 33.28, 39.17, 56.26 (2C), 60.93, 106.23 (2C), 126.91, 127.02, 128.61, 133.61, 134.10, 134.33, 134.60, 135.47, 139.14, 140.19, 144.62, 153.16, 162.81, 166.57, 178.67, 182.01, 183.84, 197.78. HRMS (M^+^): *m/z* calcd for C_26_H_21_NO_6_: 443.13689; found 443.13670.

*6-(Furan-2-yl)-3,4-dihydrobenzo[j]phenanthridine-1,7,12(2H)-trione* (**7c**). Prepared from **4c** (50 mg; 0.20 mmol), enaminone **5** (87.44 mg; 0.79 mmol), Ag_2_O (182.32mg; 0.79 mmol); (1 h, 17.49 mg, 26%), yellow solid m.p.: 190–191 °C. ^1^H-NMR (400 MHz, CDCl_3_): δ 2.30 (q, 2H, *J* = 6.7 Hz, 3-H), 2.95 (t, 2H, *J* = 6.7 Hz, 2-H), 3.20 (m, 2H, *J* = 6.7 Hz, 4-H), 6.64 (m, 1H, 4′ -H), 7.26 (m, 1H, 3′ - or 5′ -H), 7.66 (m, 1H, 5′ - or 3′ -H), 7.81 (m, 2H, 8- and 9-H), 8.13 (m, 2H, 7- and 10-H); ^13^C-NMR (100 MHz, CDCl_3_): δ 21.50, 33.28, 39.19, 106.23, 112.17, 115.03, 126.89, 127.01, 127.37, 127.89 133.91, 134.14, 134.33, 134.51, 134.60, 145.25, 153.15, 166.62, 166.84, 182.05, 197.54. HRMS (M^+^): *m/z* calcd for C_21_H_13_NO_4_: 343.08446; found 343.08449. 

*6-(Thiophen-2-yl)-3,4-dihydrobenzo[j]phenanthridine-1,7,12(2H)-trione* (**7d**). Prepared from **4d** (50 mg; 0.19 mmol), enaminone **5** (123.38 mg; 1.11 mmol), Ag_2_O (257.49 mg; 1.11 mmol); (5 h, 63.15 mg, 95%), yellow solid m.p.: 224–226 °C. ^1^H-NMR (400 MHz, CDCl_3_): δ 2.28 (q, 2H, *J* = 6.7 Hz, 3-H), 2.92 (t, 2H, *J* = 6.7 Hz, 2-H), 3.16 (t, 2H, *J* = 6.7 Hz, 4-H), 7.15 (m, 1H, 4′ -H), 7.58 (m, 1H, 3′ - or 5′ -H), 7.74 (m, 1H, 5′ - or 3′ -H), 7.81 (m, 2H, 8- and 9-H), 8.13 (m, 1H, 7- or 10-H), 8.16 (m, 1H, 10- or 7-H); ^13^C-NMR (100 MHz, CDCl_3_): δ 21.49, 33.10, 39.20, 124.85, 126.71, 127.13, 127.79, 130.86, 131.67, 133.98, 134.22, 134.49, 142.07, 145.40, 154.16, 155.12, 160.08, 166.52, 182.54, 183.86, 197.58. HRMS (M^+^): *m/z* calcd for C_21_H_13_NO_3_S: 359.06161; found 359.06138.

*6-(Thiophen-3-yl)-3,4-dihydrobenzo[j]phenanthridine-1,7,12(2H)-trione* (**7e**). Prepared from **4e** (50 mg; 0.19 mmol), enaminone **5** (82.19 mg; 0.74 mmol), Ag_2_O (171.39 mg; 0.74 mmol), (7 h, 10.5 mg, 16%), yellow solid m.p.: 208–210 °C. ^1^H-NMR (400 MHz, CDCl_3_): δ 2.29 (q, 2H, *J* = 6.6 Hz, 3-H), 2.94 (t, 2H, *J* = 6.6 Hz, 2-H), 3.19 (t, 2H, *J* = 6.6 Hz, 4-H), 7.26 (m, 1H, 4′ - or 5′ -H), 7.39 (m, 1H, 5′ - or 4′ -H), 7.80 (m, 3H, 2′ -, 8- and 9-H), 8.14 (m, 2H, 7- and 10-H). ^13^C-NMR (100 MHz, CDCl_3_): δ 21.59, 33.23, 39.18, 124.87, 124.97, 126.84, 127.11, 127.62 (2C), 128.68, 133.66, 134.06, 134.24, 134.51, 140.76, 144.77, 157.61, 166.93, 182.24, 183.83, 197.81. HRMS (M^+^): *m/z* calcd for C_21_H_13_NO_3_S: 359.06161; found 359.06138.

*6-(1H-Pyrrol-2-yl)-3,4-dihydrobenzo[j]phenanthridine-1,7,12(2H)-trione* (**7f**). Prepared from **4f** (50 mg; 0.20 mmol), enaminone **5** (87.75 mg; 0.79 mmol), Ag_2_O (182.97 mg; 0.79 mmol) (1 h, 7.43 mg, 11%), yellow solid m.p.: 230–232 °C. ^1^H-NMR (400 MHz, CDCl_3_) δ: 2.26 (q, 2H, *J* = 6.6 Hz, 3-H), 2.88 (t, 2H, *J* = 6.8 Hz, 2-H), 3.10 (t, 2H, *J* = 6.3 Hz, 4-H), 6.41 (m, 1H, 4′ -H), 7.10 (m, 1H, 3′ - or 5′ -H), 7.53 (m, 1H, 5′ - or 3′ -H), 7.79 (m, 2H, 9- and 10-H), 8.09 (m, 1H, 8 or 11-H), 8.21 (m, 1H, 11 or 8-H). ^13^C-NMR (100 MHz, CDCl_3_) δ: 20.43, 32.49, 38.21, 110.66, 118.09, 121.14, 122.39, 124.56, 125.33, 126.33, 129.66, 132.97, 133.13, 133.34, 133.39, 146.26, 150.47, 165.88, 188.04, 183.97, 196.61. HRMS (M^+^): *m/z* calcd for C_21_H_14_N_2_O_3_: 342.10044; found 342.09967.

*2,4-Dimethyl-6-phenylbenzo[g]pyrimido[4,5-c]isoquinoline-1,3,7,12(2H,4H)-tetraone* (**8a**). Prepared from **4a** (50 mg; 0.19 mmol), aminouracil **6** (80.11 mg; 0.57 mmol), Ag_2_O (131.74 mg; 0.57 mmol (5 h, 60.7 mg, 81%), yellow solid, m.p.: 261–263 °C. ^1^H-NMR (400 MHz, CDCl_3_): δ 3.53 (s, 3H, 4-*N*Me), 3.79 (s, 3H, 2-*N*Me), 7.54 (m, 5H, arom.), 7.79 (m, 2H, 8- and 9-H), 8.07 (m, 1H, 7- or 10-H), 8.15 (m, 1H, 10- or 7-H). ^13^C-NMR (100 MHz, CDCl_3_): δ 29.36, 30.60, 106.18, 123.20, 126.78, 127.31, 128.27 (2C), 129.28 (2C), 130.18, 133.61, 134.55, 135.33, 135.44, 139.77, 149.99, 151.31, 152.59, 158.80, 165.98, 180.86, 184.46. HRMS (M^+^): *m/z* calcd for C_23_H_15_N_3_O_4_: 397.10626; found 397.10675. 

*2,4-Dimethyl-6-(3,4,5-trimethoxyphenyl)benzo[g]pyrimido[4,5-c]isoquinoline-1,3,7,12(2H,4H)-tetraone* (**8b**). Prepared from **4b** (50 mg; 0.14 mmol), aminouracil **6** (79.65 mg; 0.56 mmol), (5 h, 47 mg, 68%), Ag_2_O (196.18 mg; 0.85 mmol), yellow solid, m.p.: 290–291 °C. IR (KBr): ν_max_ cm^–1^: 1673 (C=O). ^1^H-NMR (400 MHz, CDCl_3_): δ 3.53 (s, 3H, 4-*N*Me), 3.79 (s, 3H, 2-*N*Me), 3.89 (s, 6H, 3′ - and 5′ -OMe), 3.97 (s, 3H, 4′ -OMe), 6.81 (s, 2H, 2′ - and 6′ -H), 7.81 (m, 2H, 8- and 9-H), 8.08 (m, 1H, 7- or 10-H), 8.16 (m, 1H, 10- or 7-H). ^13^C-NMR (100 MHz, CDCl_3_): δ 29.18, 30.40, 56.37 (2C), 61.09, 105.86, 106.99 (2C), 123.03, 126.60, 126.99, 133.56, 134.41 (2C), 134.65, 135.12, 140.07, 150.05, 151.12, 152.27, 152.99 (2C), 158.56, 165.24, 180.70, 184.32. HRMS (M^+^): *m/z* calcd for C_26_H_21_N_3_O_7_: 487.13795; found 487.13926.

*6-(Furan-2-yl)-2,4-dimethylbenzo[g]pyrimido[4,5-c]isoquinoline-1,3,7,12(2H,4H)-tetraone* (**8c**). Prepared from **4c** (50 mg; 0.20 mmol), aminouracil **6** (111.04 mg; 0.79 mmol), Ag_2_O (182.32 mg; 0.79 mmol), (1.5 h, 55.6 mg, 73%) yellow solid, m.p.: 214–216 °C. ^1^H-NMR (400 MHz, CDCl_3_): δ 3.51 (s, 3H, 4-*N*Me), 3.79 (s, 3H, 2-*N*Me), 6.67 (m, 1H, 4′ -H), 7.38 (m, 1H, 3′ - or 5′ -H), 7.68 (m, 1H, 5′ - or 3′ -H), 7.81 (m, 2H, 8- and 9-H), 8.15 (m, 2H, 7- and 10-H). ^13^C-NMR (100 MHz, CDCl_3_): δ 29.14, 30.33, 105.60, 112.41, 116.72 (2C), 126.65, 126.99, 133.68, 134.22 (2C), 134.35, 134.95, 146.00 (2C), 151.84, 152.42, 158.47, 164.05, 180.37, 183.88. HRMS (M^+^): *m/z* calcd for C_21_H_13_N_3_O_5_: 387.08552; found 387.08519.

*2,4-Dimethyl-6-(thiophen-2-yl)benzo[g]pyrimido[4,5-c]isoquinoline-1,3,7,12(2H,4H)-tetraone* (**8d**). Prepared from **4d** (50 mg; 0.19 mmol), aminouracil **6** (78.41mg; 0.55 mmol) and Ag_2_O (128.79 mg; 0.55 mmol), (3 h, 60.5 mg, 81%) yellow solid, m.p.: 263–265 °C. ^1^H-NMR (400 MHz, CDCl_3_): δ 3.50 (s, 3H, 4-*N*Me), 3.79 (s, 3H, 2-*N*Me), 7.18 (m, 1H, 4′ -H), 7.64 (m, 1H, 3′ - or 5′ -H), 7.81 (m, 2H, 8- and 9-H), 8.01 (m, 1H, 5′ - or 3′ -H), 8.13 (m, 1H, 7- or 10-H), 8.17 (m, 1H, 10- or 7-H). ^13^C-NMR (100 MHz, CDCl_3_): δ 29.09, 30.37, 105.32, 121.96, 126.36, 127.18, 128.21, 132.52, 133.66, 133.79, 134.30, 134.37, 134.86, 142.17, 150.98, 151.09, 152.03, 157.04, 158.48, 180.95, 184.47. HRMS (M^+^): *m/z* calcd for C_21_H_13_N_3_O_4_S: 403.06268; found 403.06174.

*2,4-Dimethyl-6-(thiophen-3-yl)benzo[g]pyrimido[4,5-c]isoquinoline-1,3,7,12(2H,4H)-tetraone* (**8e**). Prepared from **4e** (50 mg; 0.19 mmol), aminouracil **6** (52.21 mg; 0.37 mmol) and Ag_2_O (257.22 mg; 1.11 mmol), (5 h, 18.5 mg, 25%) yellow solid, m.p.: 280–282 °C. ^1^H-NMR (400 MHz, CDCl_3_): δ 3.52 (s, 3H, 4-*N*Me), 3.79 (s, 3H, 2-*N*Me), 7.35 (m, 1H, 4′ - or 5′ -H), 7.40 (m, 1H, 5′ - or 4′ -H), 7.81 (m, 2H, 8- and 9-H), 7.94 (m, 1H, 2′ -H), 8.14 (m, 2H, 7- and 10-H). ^13^C-NMR (100 MHz, CDCl_3_): δ 29.17, 30.36, 105.78, 124.84, 126.54, 127.10, 129.07, 129.49, 133.52, 134.38 (2C), 134.83, 135.05, 140.28, 151.13, 152.51 (2C), 158.57, 159.61, 180.85, 184.38. HRMS (M^+^): *m/z* calcd for C_21_H_13_N_3_O_4_S: 403.06268; found 403.06184.

*2,4-Dimethyl-6-(1H-pyrrol-2-yl)benzo[g]pyrimido[4,5-c]isoquinoline-1,3,7,12(2H,4H)-tetraone* (**8f**). Prepared from **4f** (50 mg; 0.20 mmol), aminouracil **6** (55.72 mg; 0.40 mmol) and Ag_2_O (228.71 mg; 0.99 mmol), (3.5 h, 29.8 mg, 39%) red solid, m.p.: 289–291 °C. ^1^H-NMR (400 MHz, DMSO-*d*_6_): δ 3.28 (s, 3H, 4-*N*Me), 3.67 (s, 3H, 2-*N*Me), 6.28 (s, 1H, 4′ -H), 7.21 (m, 2H, 3′ - or 5′ -H), 7.92 (m, 2H, 8 or 9H), 7.97 (m, 1H, 7- or 10-H), 8.10 (m, 1H, 10- or 7-H), 11.67 (bs, 1H, NH). ^13^C-NMR (100 MHz, CDCl_3_) δ: 29.29, 30.81, 104.32, 111.15, 119.27, 121.09, 125.84, 126.13, 127.73, 130.30, 134.69, 135.00, 135.16, 135.30, 151.12, 151.86, 152.94, 153.96, 159.01, 181.38, 185.39. HRMS (M^+^): *m/z* calcd for C_21_H_14_N_4_O_4_: 386.10150; found 386.10104.

### 3.2. Anticancer Assay

The cell lines used in this work were obtained from the American Type Culture Collection (ATCC, Manasas, VA, USA). They included MRC-5 normal human lung fibroblasts (CCL-171), AGS human gastric adenocarcinoma cells (CRL-1739), SK-MES-1 human lung cancer cells (HTB-58) and J82 human bladder carcinoma cells (HTB-1). After the arrival of the cells, they were proliferated in the corresponding culture medium as suggested by the ATCC. The cells were stored in medium containing 10% glycerol in liquid nitrogen. The viability of the cells after thawing was higher than 90%, as assessed by trypan blue exclusion test. Cells were sub-cultured once a week and the medium was changed every two days. Cells were grown in the following media: MRC-5, SKMES-1, and J82 in MEM and AGS cells in Ham F-12. The MEM medium contained 2 mM l-glutamine, 1 mM sodium pyruvate and 1.5 g/L sodium hydrogen carbonate. Ham F-12 was supplemented with 2 mM l-glutamine and 1.5 g/L sodium hydrogen carbonate. All media were supplemented with 10% heat-inactivated FBS, 100 IU/mL penicillin and 100 μg/mL streptomycin in a humidified incubator with 5% CO_2_ in air at 37 °C. For the experiments, cells were plated at a density of 50.000 cells/mL in 96-well plates. One day after seeding, the cells were treated with the medium containing the compounds at concentrations ranging from 0 up to 100 μM during 3 days and finally the MTT reduction assay was carried out. The final concentration of MTT was 1 mg/mL. The compounds were dissolved in DMSO (1% final concentration) and complete medium. Untreated cells (medium containing 1% DMSO) were used as controls. Each experiment was carried out in sextuplicate.

## 4. Conclusions

We have developed the synthesis of a variety of 6-aryl-substituted benzo[*j*]phenanthridine- and benzo[*g*]pyrimido[4,5-*c*]isoquinolinequinones from 1,4-naphthoquinone, arylaldehydes, aminocyclo- hexenone and aminouracil *via* an efficient one-step procedure to assemble the respective ABCD-ring systems. The compounds thus obtained were tested against normal human lung fibroblasts (MRC-5) and on gastric adenocarcinoma (AGS), lung cancer (SK-MES-1), and bladder carcinoma (J82) cell lines. Structure-activity relationships within these series of angular quinones reveal that insertion of a pyrrol-2-yl group at the 6-position of the benzo[*j*]phenanthridinequinone scaffold (*i.e*., compound **7f**) and insertion of a furan-2-yl group in the 6-position (compound **8c**) of the benzo[*g*]pyrimido[4,5-*c*]isoquinolinequinone scaffold are more significant to increase the potency of these pharmacophores against human gastric adenocarcinoma cell line. Compound **8c** is the most promising member because of its potency and high selectivity (9.6) on the human gastric adenocarcinoma cell line.

## Figures and Tables

**Table 1:** Preparation of benzo[*j*]phenanthridine- and benzo[*g*]pyrimido[4,5-c]isoquinolinequinone derivatives.

Acylnaphthohydroquinone	Enaminone	Product	N°	Yield (%)
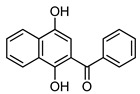 **4a**	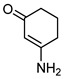 **5**	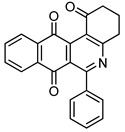	**7a**	45
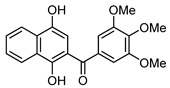 **4b**	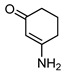 **5**	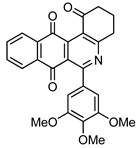	**7b**	93
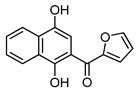 **4c**	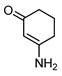 **5**	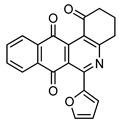	**7c**	26
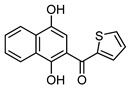 **4d**	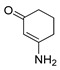 **5**	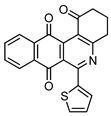	**7d**	95
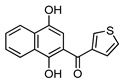 **4e**	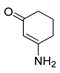 **5**	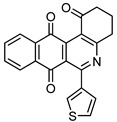	**7e**	16
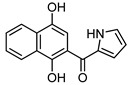 **4f**	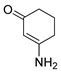 **5**	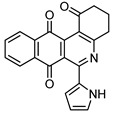	**7f**	11
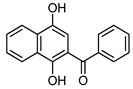 **4a**	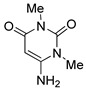 **6**	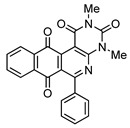	**8a**	81
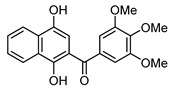 **4b**	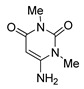 **6**	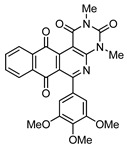	**8b**	68
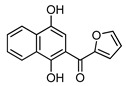 **4c**	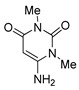 **6**	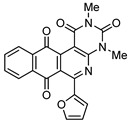	**8c**	73
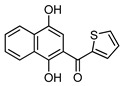 **4d**	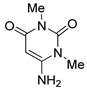 **6**	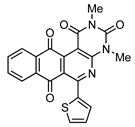	**8d**	81
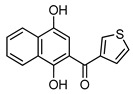 **4e**	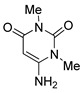 **6**	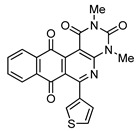	**8e**	25
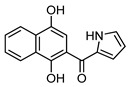 **4f**	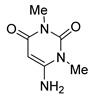 **6**	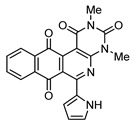	**8f**	39

**Table 2:** Cytotoxic activity of benzo[*j*]phenanthridine- and benzo[*g*]pyrimido[4,5-*c*]isoquinolinequinone derivatives.

		IC_50_ ± SEM ^a^ (µM)			
Compound	N°	MRC-5 ^b^	AGS ^c^	SK-MES-1 ^d^	J82 ^e^
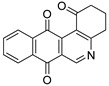	**1**	41.3 ± 2.2	19.0 ± 0.9	51.6 ± 2.7	62.6 ± 3.4
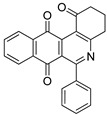	**7a**	35.4 ± 2.8	21.4 ± 1.3	>100	99.4 ± 6.6
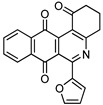	**7c**	>100	42.4 ± 2.3	>100	>100
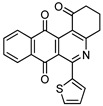	**7d**	14.3 ± 1.2	40.5 ± 2.6	64.4 ± 3.2	59.7 ± 2.5
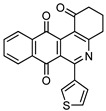	**7e**	32.3 ± 2.6	47.8 ± 2.5	>100	>100
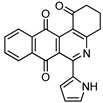	**7f**	21.3 ± 1.1	10.1 ± 0.8	>100	>100
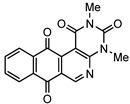	**2**	72.8 ± 3.4	82.1± 4.4	67.5 ± 3.6	78.6 ± 3.9
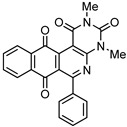	**8a**	30.3 ± 3.1	4.6 ± 0.2	12.4 ± 1.2	5.0 ± 0.3
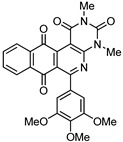	**8b**	61.1 ± 5.4	36.1 ± 2.0	85.8 ± 5.1	>100
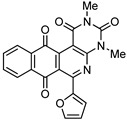	**8c**	31.8 ± 2.5	3.3 ± 0.2	>100	>100
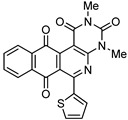	**8d**	>100	19.6 ± 1.4	>100	40.3 ± 3.3
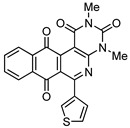	**8e**	59.1 ± 4.6	18.5 ± 1.3	59.8 ± 4.5	23.4 ± 1.1
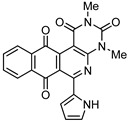	**8f**	77.8 ± 5.3	>100	74.1 ± 5.8	>100
etoposide	-	3.9 ± 0.21	0.36 ± 0.15	2.8 ± 0.18	0.80 ± 0.04

^a^ Data represent mean average values for six independent determinations; ^b^ Normal human lung fibroblasts cells; ^c^ Human gastric adenocarcinoma cell line; ^d^ Human lung cancer cell line; ^e^ Human bladder carcinoma cell line.
